# Diagnostic value of plasma and bronchoalveolar lavage samples in acute lung allograft rejection: differential cytology

**DOI:** 10.1186/s12931-016-0391-y

**Published:** 2016-06-21

**Authors:** Nicole E. Speck, Macé M. Schuurmans, Christian Murer, Christian Benden, Lars C. Huber

**Affiliations:** Division of Pulmonology, University Hospital Zurich, Rämistrasse 100, CH-8091, Zurich, Switzerland

**Keywords:** Blood, Bronchoalveolar lavage, Cytology, Diagnosis, Graft rejection, Lung transplantation, Plasma

## Abstract

Diagnosis of acute lung allograft rejection is currently based on transbronchial lung biopsies. Additional methods to detect acute allograft dysfunction derived from plasma and bronchoalveolar lavage samples might facilitate diagnosis and ultimately improve allograft survival. This review article gives an overview of the cell profiles of bronchoalveolar lavage and plasma samples during acute lung allograft rejection. The value of these cells and changes within the pattern of differential cytology to support the diagnosis of acute lung allograft rejection is discussed. Current findings on the topic are highlighted and trends for future research are identified.

## Background

Lung transplantation is an established treatment option for selected patients with advanced lung disease [1, 2]. However, despite improvement in surgical, postoperative and immunosuppressive management, the overall survival after lung transplantation remains lower than for recipients of other solid organ transplants [2, 3]. This is mainly due to development of chronic lung allograft dysfunction (CLAD), of which bronchiolitis obliterans syndrome (BOS) is the most common phenotype, being observed in more than 75 % of lung transplant recipients after 10 years [4, 5].

A major risk factor for the development of CLAD is the occurrence of repeated episodes of higher grade acute lung allograft rejection (AR) [6–8]. Acute lung AR, together with infections that by itself might trigger AR, is one of the most common complications occurring early after lung transplantation and affects more than 30 % of adult lung transplant recipients within the first year post transplantation [2].

Acute lung AR is suggested by a decline of lung function that is not explained by other reasons such as infection, left-sided heart failure or weight gain. The gold standard for AR diagnosis is the analysis of serial transbronchial lung biopsies (TBB) [9]. This method, however, is invasive and carries the risk of pulmonary bleeding and pneumothorax [10, 11]. Moreover, interobserver variability and sampling error limit the reliability of this method [12, 13].

Various attempts have been undertaken to validate alternative diagnostic methods, including clinical and/or radiological criteria. However, these patterns only allow delayed and retrospective diagnosis [14] and are of limited sensitivity [14, 15]. Moreover, cough, dyspnoea, low-grade fever, perihilar infiltrates and deterioration of pulmonary function may appear in other common conditions after transplantation and do not distinguish AR from infection [14, 16].

Alternatively, alterations in bronchoalveolar lavage (BAL) and plasma samples have been examined for specific changes during acute lung AR. Sampling cells by BAL bronchoscopy is less invasive than TBB and allows for repetitive harvesting. Although rare, complications of BAL have been observed and include fever, wheezing or bleeding [17]. Being able to diagnose acute lung AR in peripheral blood would be desirable for various reasons. Peripheral blood is easily accessible and complications are rare. However, peripheral blood may not reliably reflect processes in the lung and might thus not reach high specificity. The lung allograft rejection gene expression observational study (LARGO) is currently analysing new non-invasive techniques to assess biomarkers in peripheral blood. The study has shown encouraging results but further research is needed [18].

**Fig. 1:**
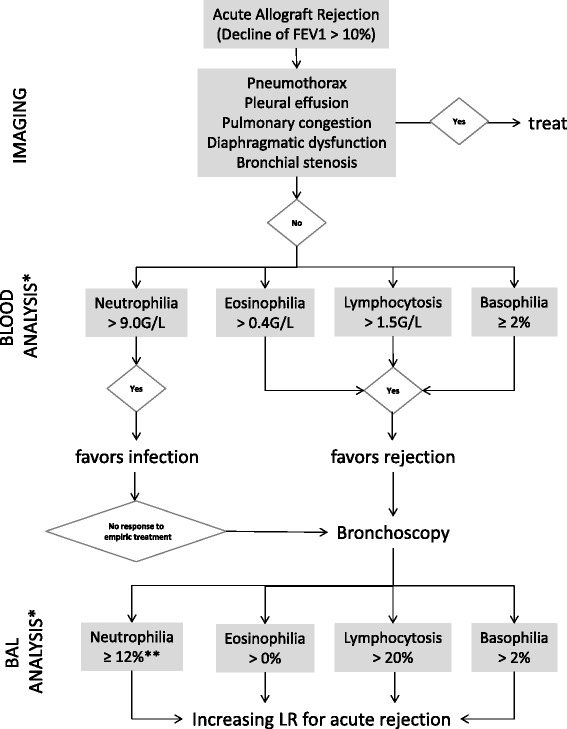
Algorithm based on serum and BAL cell count and analysis. This descriptive algorithm attempts to describe probabilities for acute AR in lung transplant recipients and as such might assist in decision-making to increase or decrease the likelihood for acute AR in the context of the clinical presentation. Since results from studies with very different designs have been included direct translation in a clinical setting is not feasible and the use of this algorithm does not obviate the need for biopsy to confirm or exclude histology-proven acute rejection. It is important to note that in the absence of an explicit allograft infection, in which bronchoscopy might be postponed in favour of empiric antimicrobial treatment, any lung transplant recipient with a lung functional drop (FEV1 > 10 %) should undergo diagnostic bronchoscopy independent of blood analysis. * Numbers may vary between different studies. ** Absence of microbiological evidence for infection

Since both BAL and plasma samples have the advantage of being fast and less invasive in comparison with TBB, we reviewed here the evidence for the use of BAL and plasma samples for detection of acute lung AR in lung transplant recipients. This article aims to provide answers to the question whether changes in cell count or percentage of cellularity appear to be specific for acute lung AR and whether they precede clinical symptoms and decline in lung function. An illustrative algorithm for the likelihood of acute AR depending on BAL and plasma cellularity is provided in Fig. 1.

More specifically, this review summarises the experimental and clinical evidence on the differential cellularity profiles in BAL and plasma samples during acute lung AR. Current findings on this topic are discussed and areas for future research are identified.

## Methods

We searched the electronic databases Medline (Bethesda, MD, USA: U.S. National Library of Medicine), EMBASE (Amsterdam, NL: Elsevier B.V.) and Web of Science Core Collection (New York, NY, USA: Thomson Reuters). Medical subject heading (MeSH) terms included „bronchoalveolar lavage fluid/cytology“ in combination with “graft rejection” and “lung transplantation” for BAL and “blood/cytology” or “plasma/cytology” in combination with “graft rejection” and “lung transplantation” for data on plasma samples. Publications were eligible if they provided information on cytological changes in BAL or peripheral blood during acute lung AR. We considered articles published in English until June 1, 2015. This included experimental studies, prospective and retrospective clinical studies, review articles and case reports. No other restrictions were applied. We then selected those articles that fulfilled our inclusion criteria. Additionally, we scanned the references of all selected articles to find additional literature that was related to our research question. Finally, 54 papers were eligible to be included in our review. An ordered list of the type and number of articles included is provided in Table 1.

**Table 1:** Types and number of references included in this review article

Content	Study design	Number of studies included	Total number of patients
Cytology	Experimental	22	
	Prospective	8	392
	Retrospective	19	1561
	Review article	3	
	Case report	2	

We then evaluated the selected articles and compiled an extensive table, listing every cell type, the reference that mentioned these parameters as well as the observed data. While writing the review article more papers were drawn on for background information. Each author reviewed the entire document and provided input before the final manuscript was completed.

### Cells in BAL and plasma samples

AR has been recognised as an interacting process of T cell subpopulations, macrophages, granulocytes and B cells [19, 20]. Cellular changes occurring during acute lung AR can be observed either directly in the allograft using biopsies or, alternatively, in bronchoalveolar lavage fluid or plasma samples.

Bronchoalveolar lavage allows recovering cells and proteins present on the epithelial surface of the lower respiratory tract [21]. Originally, BAL fluid was the primary method to identify lung infections in immunosuppressed patients since viral, bacterial, fungal and protozoal infections are detected with high sensitivity and specificity [17, 22]. Achterrath and colleagues were the first to look at BAL fluid for the study of differential cytology during acute lung AR, using a canine model [23]. Since then, various investigators have examined cellular changes in BAL samples during acute lung AR. Moreover, in the context of prognosis, the presence of eosinophils in BAL (≥2 %) has been associated with the development of CLAD and reduced overall survival [24]. Some lung transplant centres routinely collect BAL samples after lung transplantation (“surveillance bronchoscopy”) [24, 25]. However, the confounder of concurrent infections and the lack of specimen standardization and equipment limit the routine use of BAL to diagnose acute AR [17].

The technique of retrieving BAL samples might vary between different centres [26]. Differences concern the means of harvesting, the discard of the first aliquot as well as the pre-analytic phase. BAL fluid is analysed for microbiological infiltrates, total cell count and cytology. The number of different cells detected is divided by the total cell count and expressed as percentages. To determine lymphocyte phenotypes flow cytometry is performed [27]. Table 2 provides an overview of the bronchoalveolar lavage fluid cell profile in different populations.

**Table 2:** Percentage of cellularity in BAL in different populations

	Basophils	Eosinophils	Neutrophils	Lymphocytes	AM	Total cell count
Healthy, non-smoking individuals [78]	<1 %	<2 %	<3 %	<10–15 %	>85 %	31–350/μl
Smokers [78, 101, 102]		<3 %	3 %	<7 %	95 %; 3–5-fold increase of total cells	80–1100/μl; 3-fold increase of total cells
Stable lung transplant recipients [17, 64]		<1 %	4–12 %	5–19 %	71–94 %	140–442/μl; increased, high variance
Acute lung AR [17, 25, 37]	≥2 %	>0 %	15–30 %	10–60 %	30–70 %	200 - > 700/μl; increased, high variance
Association with AR						
- Direction	↑	↑	↑	↑	↓	↑
- Replicability	moderate	moderate	high	high	high	

For this review article, we selected cells that are routinely analysed in clinical diagnostics, including neutrophils, lymphocytes, eosinophils, basophils, monocytes and alveolar macrophages (AM). Natural killer cells (NK cells) and B lymphocytes (B cells) as subgroups of lymphocytes were considered separately. In order to keep this paper close to clinical practice, T cell subgroups such as CD25^+^ regulatory cells, CD8+ T cells or T cells with other clusters of differentiation have not been included in this review article although findings have emerged on these recently [28–30]. Tables 3 and 4 provide a list of cells studied with the observed direction of change during acute lung AR and the respective references.

**Table 3:** Observed pattern of cells in BAL samples during acute lung AR

Cell	BAL	Reference	Sensitivity	Specificity	Cut-off
Basophils	↑	[37, 38]			
	↑ (rare)	[25]			
Eosinophils	↑	[59] (experimental)			
	↑	[27, 37, 38, 41, 43]			
	↑ (rare)	[25]			
	0	[32, 70]			
	*	[42]			
Neutrophils	↑	[32, 37–40]	74 %	82 %	≥12 %
	0	[42, 43]			
Lymphocytes	↑	[47–49] (experimental)			
	↑	[14, 25, 27, 32, 37, 38, 42, 50, 51]	64 % 65 % 40 %	77 % 92.5 % 96 %	≥15 % >20 % ≥20 %
	↑0	[40]			
NK cells	↓	[25, 85]			
B cells	↑ (rare)	[25]			
	↑0	[85]			
Monocytes	↓	[25]			
Macrophages	↑	[47] (experimental)			
	↓	[27, 32, 40–42, 50]			

0, No correlation

↑, Increased

↑0, Increased, but statistically not relevant

↓, Decreased

*, Not detected

(rare), Rarely detected

**Table 4:** Observed patterns of cells in plasma samples during acute lung AR

Cell	Plasma	Reference	Sensitivity	Specificity	Cut-off
Basophils	↑	[37, 38]	42 %	94 %	≥2 %
Eosinophils	↑	[45]	72 %	75 %	Increase ≥ 9 cells/μl
Neutrophils	0	[45]			
Lymphocytes	↑	[37, 38]			
	0	[40]			
NK cells					
B cells					
Monocytes					
Macrophages					

0, No correlation

↑, Increased

### Neutrophils

Neutrophils are an integral part of innate immunity and constitute the largest portion of circulating white blood cells [31]. They exhibit phagocytic functions and secrete toxic granules containing granzyme B, perforin and myeloperoxidase, thus, playing a major role in anti-microbial defence [31]. They are rapidly drawn to sites of inflammation, apparently by the chemotactic factors IL-8 and IL-17 [32]. Neutrophil defence mechanisms have been associated with several conditions in the lung including viral infections (e.g. Influenza and RSV) [33, 34].

In the context of BAL fluid analysis neutrophils have emerged as key mediators of a recently identified CLAD phenotype, which has been defined as neutrophilic reversible allograft dysfunction (NRAD). NRAD is characterised by BAL neutrophilia (≥15 %) without any other signs for infection. These patients respond well to long-term macrolide therapy [35]. A recent study, however, has challenged this NRAD phenotype since response to azithromycin was found to be independent of neutrophil count [36].

Clinical data on neutrophils during acute lung AR point toward an increase in neutrophils in BAL samples [32, 37–40]. A prospective study with 20 lung or heart-lung transplant recipients found increased numbers of neutrophils during rejection episodes that occurred after post-operative day 180. Furthermore, BAL neutrophilia ≥ 12 % after post-operative day 31 diagnosed acute AR with a specificity of 82 % and a sensitivity of 74 %. Even though this change is not specific for rejection alone, increased neutrophils in BAL should be considered suspicious for rejection when occurring after the first postoperative month [37, 38, 40].

Moreover, neutrophils in BAL have been shown to correlate with severity of acute AR in a number of studies [25, 32, 41]. However, one should remind that the broad use of macrolides complicates interpretations of BAL neutrophilia between current studies and studies performed before the diffusion of macrolides less than a decade ago.

In contrast to these findings, other studies found no association between neutrophil percentage and acute lung AR [42, 43] or activation of neutrophils during episodes of rejection [43].

In addition to these observational data, a gene-based diagnostic classifier for acute lung AR has been proposed. Patil and colleagues found that increased BAL neutrophil gene expression was associated with acute AR. The number of neutrophils in biopsies also increased during rejection episodes, yet this characteristic was no better discriminator than the gene-based classifier [44].

Little data exists on blood neutrophils during acute AR. In one prospective study blood neutrophil count was not associated with acute lung or cardiac allograft rejection. Yet, a lacking increase of neutrophils in the presence of raised eosinophils was considered a specific and early sign for clinically relevant rejection [45].

**Table:** 

Early BAL neutrophilia in absence of an infection should raise suspicion for acute lung AR.	

### Lymphocytes

Lymphocytes are mediators of the adaptive and innate immune system and consist of several subtypes including T lymphocytes (T cells), B cells and NK cells. Most studies focusing on lymphocytes make conclusions on lymphocytes in general and do not address the subtypes. T cells have been identified as the most important mediators of acute AR and are thus of special interest in transplantation medicine [46].

Experimental data points towards an increase in BAL lymphocytes during acute lung AR: Data obtained from an experimental rejection model using rats [47, 48] and dogs [49] described an increase in the number of BAL T lymphocytes few days after onset of AR [47, 48]. Moreover, the increase of BAL lymphocytes was more pronounced with higher grades of rejection [49].

In a clinical setting, all reviewed articles found increased BAL lymphocyte counts during acute AR, however at different time points [32, 37, 38, 42]. Lymphocytes have been reported to be elevated (≥15 %) during the first postoperative month, with a specificity of 77 % and sensitivity of 64 % (*p* < 0.05) to diagnose acute AR. Specificity for lymphocytes for infection was higher during months 1 to 6 (86 %), while sensitivity remained unchanged [37, 38]. In a prospective study, BAL lymphocytes were found to be higher during grade A2 mild acute AR compared with matched controls at a median sampling time of seven months [42].

However, increased levels of BAL lymphocytes show an insufficient sensitivity to diagnose acute AR [14, 50, 51]. BAL lymphocytosis of ≥ 20 % was very specific (96 %) but not sensitive (40 %) to diagnose acute AR [14].

Increased BAL lymphocytes are not exclusively found in acute AR but have been observed during other post-transplant complications [27, 40]. More specifically, BAL lymphocyte percentage was found elevated during acute AR (19 % ± 5.6), infection (22.5 % ± 4.5) and in CLAD (29.5 % ± 7.9), although these findings did not reach significance [40]. Another research group investigating acute AR and infection observed the highest number of lymphocytes during episodes of rejection. In this study, BAL lymphocyte percentage > 20 % diagnosed acute AR with a specificity of 92.5 % and a sensitivity of 65 % (positive predictive value (PPV) 79.5 %, negative predictive value (NPV) 85.4 %). However, in 11 % of BAL samples with significantly increased BAL lymphocytes infection was identified (four of five patients had CMV pneumonitis) [27]. In one of the first prospective studies comparing BAL and TBB cell profiles mean BAL lymphocyte count was significantly higher in acute AR than in infection, treated acute AR and CLAD. However, distinguishing between these conditions based on lymphocytes levels was not possible due to considerable overlap between the groups. Lymphocyte percentage exceeded the upper level of normal (15 %) in 23 % of BAL samples with acute AR, but also in 13 % of samples with infection. Although rarely seen, BAL lymphocytosis of > 25 % was suggestive for the diagnosis of acute AR [50]. Greenland and colleagues calculated the odds for having rejection rather than infection per standard deviation increase of lymphocytes in BAL samples with either infection or biopsy-confirmed rejection grade ≥ A1. Raised percentages of lymphocytes (>12 %) turned out to be associated with greater odds (OR = 2.04) of rejection [25].

The correlation between BAL lymphocyte count and rejection grade in TBB show inconsistent findings. Several studies found no association between lymphocytosis in BAL and histopathological rejection grade [27, 50, 51] whereas others described a correlation between BAL lymphocyte count and grade of acute AR in biopsy specimens [14, 25, 32].

Data about lymphocytes in peripheral blood differs considerably between studies [37, 38, 40]. Blood lymphocytes were raised significantly during acute AR between day 31 and day 180 after transplantation compared to controls with neither rejection nor infection [37, 38]. In contrast, peripheral blood lymphocytes did not change significantly in another study [40].

**Table:** 

High BAL lymphocyte counts are associated with acute lung AR but are also found in other complications following lung transplantation. In most studies, BAL lymphocytosis showed an acceptable specificity for AR. Sensitivity, however, is low.	

### Eosinophils

Eosinophils are bone marrow-derived granulocytes and account for less than 5 % of circulating leukocytes. Upon stimulation by interleukins (IL) such as IL-4, IL-5 and IL-13 eosinophils produce reactive oxygen species and toxic granule proteins (e.g. eosinophil cationic protein and major basic protein) [52]. Eosinophils have been associated with various pulmonary conditions such as asthma, eosinophilic granulomatosis with polyangiitis (“Churg-Strauss syndrome”), drug reactions, helminthic infections, hypereosinophilic syndrome as well as acute and chronic eosinophilic pneumonia [16, 53–56]. More specifically, eosinophils have been shown to damage the lung by degrading connective tissue and injuring epithelial and micro-vascular structures [57, 58].

Raised BAL eosinophils were found to be a marker of early lung AR in rats [59]. This result is in line with previous experimental data where raised eosinophils in blood and allograft were associated with the rejection process in other solid organs including the kidney, liver and heart [60–63].

Increased numbers of eosinophils in peripheral blood precede clinically significant AR in both pulmonary and cardiac transplant recipients. This was first shown in a retrospective study with 58 pulmonary and 56 cardiac allograft recipients. The mean maximum eosinophil count in the three days before treated lung AR was 140/μl, which was significantly higher than when rejection or infection were absent. An increase in blood eosinophils of ≥ 9/μl in allograft recipients had a sensitivity of 72, a specificity of 75 and a modest PPV of 51 % for the detection of acute AR during postoperative month one. Trull and co-workers therefore suggested measuring blood eosinophils daily during the first postoperative month to detect acute AR at an early stage [45].

Eosinophils have appeared in BAL fluid of patients with good outcome after lung transplantation, making up < 1 % of total cells [64]. Several clinical studies have found an association between increased eosinophils in BAL and acute lung AR [25, 27, 37, 38, 43]. Greenland and co-workers found significantly raised eosinophils during acute AR. However, eosinophils were detected in < 10 % of samples. Applying a multivariate, linear regression model the authors identified eosinophils > 0 % as one of four high-risk features for rejection (besides monocytes < 75, NK cells < 5 and CD25^+^ cells > 8 %). If all of these features were absent, acute AR was unlikely with a NPV of > 96 % [25]. Besides, levels of cytotoxic eosinophil cationic protein were found to be increased in transplant recipients during acute AR or infection, which points towards activation of eosinophils in these conditions [43, 65, 66].

A relation between the amount of eosinophils in BAL and severity of acute AR has been reported [41, 67]. In a retrospective study performed in a large cohort, Vos and colleagues found that raised eosinophils in BAL fluid correlated with increasing grade A severity in biopsies with grade A rejection alone or combined A and B rejection [41].

Moreover, eosinophilia ≥ 2 % in BAL seems to correlate with worse outcome after lung transplantation [24]. In a multivariate model Verleden and colleagues identified BAL eosinophilia as the most important risk factor for CLAD development and overall mortality. The investigators indicated that acute rejection with detectable eosinophils in BAL might constitute a different form of acute AR. This phenotype might respond less to treatment and may thus be associated with worse outcome [24]. This hypothesis could also explain the observation that eosinophilia tends to be associated with more aggressive episodes of acute AR [68]. Alternatively, altered antioxidant defence might render transplant recipients more susceptible for eosinophil cytotoxic agents, as suggested by Riise and colleagues [69].

Not all investigators reported increased BAL eosinophils during acute lung AR [32, 42, 70]. A retrospective study found no statistically significant difference between eosinophil percentage in BAL fluid in patients with acute AR and patients without AR or infection. However, BAL samples of transplant recipients contained more eosinophils than those of non-transplanted controls during both event-free episodes and acute AR [70]. In another study, eosinophil number and percentage were low and hardly variable (median 0 %, interquartile range (IQR) 0–0.8 %) during acute AR [32].

Several mechanisms for the pathogenesis and activation of eosinophils during acute AR exist. Eosinophils injure the graft by releasing cytotoxic agents such as eosinophil cationic protein [65, 68]. Activation of eosinophils by helper T lymphocytes through IL-3 and IL-5 and by macrophages through IL-1 has been suggested [16, 71, 72]. However, Bewig and colleagues found no correlation between lymphocytes expressing IL-5 and the number of eosinophils in BAL [16]. This group also found that patients with BAL eosinophilia during acute AR responded well to steroids, which was confirmed in other studies [16, 73].

Eosinophils in allografts are not confined to AR but have been found in transplant recipients with viral, fungal and bacterial infection [68]. High eosinophils in BAL are indicative of acute AR if four conditions are fulfilled: Eosinophilia should be temporary, accompanied by clinical signs of rejection, in the absence of infection and respond to anti-rejection therapy [16].

**Table:** 

Eosinophils are rarely present in BAL; however, if detected and elevated in BAL and if other causes are excluded, suspicion for acute AR should be raised. Peripheral blood eosinophilia might indicate clinically significant rejection. Blood differential cellularity should be examined regularly.	

### Basophils

Basophils are circulating granulocytes representing less than 1 % of blood leukocytes in steady-state conditions of non-transplanted individuals [74]. Upon stimulation by inflammatory signals they rapidly expand within the bone marrow and are distributed to the blood, spleen and liver [75]. Invasion of the lungs has also been observed and might be of major importance in the context of allograft dysfunction and acute AR [75]. While the exact function of basophils remains unknown to date, recent evidence suggests that these cells play a critical role in a variety of immunologic disorders [76].

The role of basophilic granulocytes in lung transplant recipients has not yet been elaborated in detail. However, mild peripheral blood basophilia and their presence in BAL fluid have been associated with acute AR in clinical studies [25, 37, 38]. As such, Tikkanen and co-workers analysed samples from peripheral blood, BAL and TBB of 20 heart and heart-lung allograft recipients with and without signs of rejection in a prospective study. To assess the cell profiles over post-transplant time course, the samples were arbitrarily divided in three groups (1–30 days; 31–180 days; >180 days). Throughout follow-up, blood basophilia of ≥ 2 % was indicating acute AR with a very high specificity (94 %). Moreover, the number of basophils was increased in BAL fluid in patients with acute AR compared to controls during the first postoperative month [37]. In a recent retrospective study similar results were obtained. This work constitutes the most extensive analysis on BAL cellularity in lung transplant recipients so far [77]. BAL fluid collected from almost all patients (317 of 356) who underwent lung transplantation at the University of California (UCSF) between 1997 and 2011 was analysed. Although rarely detected in BAL samples, the presence of basophils was associated with AR [25], confirming the role of basophils as a potential surrogate marker of AR.

**Table:** 

Mild peripheral blood basophilia and their presence in BAL fluid have been associated with acute AR in clinical studies.	

### Monocytes and alveolar macrophages

Monocytes are the largest cells in blood and account for 3–8 % of all blood cells. Upon recruitment to different tissues, monocytes undergo maturation into macrophages [25]. Alveolar macrophages account for > 85 % of cells retrieved in BAL fluid of healthy non-transplanted individuals [78]. They play an important role in anti-microbial defence, inflammatory and immune reactions as well as protecting lung tissue from protease attack [21].

BAL inflammatory macrophages were raised in an experimental rejection model using rats. While acute AR occurred in all allotransplants on day two after transplantation macrophage number and percentage increased later in the rejection process, on day six [47].

Results on BAL macrophages are not consistent. AM percentage in BAL shows a trend towards reduction during acute AR [25, 32, 40, 42]. These findings, however, are not specific and are also observed during other post-transplant complications such as infection and BOS [27, 50]. BAL macrophage percentage was reduced in infection and both acute AR grade I (47.8 % ± 14.2) and grade II-III (42.7 % ± 9.9) as compared to normal controls (72.8 % ± 4.4) [40]. Similarly, BAL macrophage percentage was significantly lower in patients with acute AR (78 %) than in matched controls (91 %) at a median sampling time of seven months [42]. The same trend was observed in another study, yet the total macrophage cell count did not differ between rejecting patients and controls. The macrophage percentage was lower during acute AR due to higher neutrophil and lymphocyte counts [32]. In a scoring system developed to identify strong and independent parameters of AR, Greenland and colleagues found that higher levels of monocytes were negatively associated with acute AR. Conversely, monocytes < 75 % correlated independently with rejection scores ≥ A1 in a multivariate, linear regression model (OR 2.41) and were identified as one of four high-risk features for rejection (see eosinophils) [25].

AM counts have also been shown to correlate inversely with the histopathological grade of rejection [41, 50]. In a prospective study, Clelland and colleagues found an association between BAL macrophage number and the severity of acute AR: A lower number of macrophages in BAL correlated with a higher grade of rejection on transbronchial biopsies [50]. This trend was also observed in a retrospective study [41].

**Table:** 

Macrophage percentage in BAL samples is reduced in rejecting patients. Since the total macrophage cell count was not found to be different this finding must be due to higher neutrophil and lymphocyte counts during acute lung AR.	

### NK cells

NK cells are a subset of lymphocytes with characteristics of both innate and adaptive immunity [79]. Their role in immunity has not been entirely understood but new views are emerging. In current understanding, NK cells are important in eliminating viral infections and neoplastic cells [79]. Moreover, they can both regulate adaptive immune responses by eliminating antigen-presenting cells and T cells [80–82] and enhance such responses via cytokines such as interferon gamma (IFN-γ) and tumour necrosis factor alpha (TNF-α) [83, 84].

Few studies analysed NK cell count during acute lung AR, however these have yielded encouraging results [25, 85]. Additionally, some studies examined NK cell activity during periods of rejection [49, 86]. The discussion of these findings is beyond the scope of this review.

NK cells have been reported to decline in BAL of lung transplant patients during episodes of acute lung AR [25, 85]. NK cell percentage was reduced in patients with acute AR compared to non-rejecting controls, however the difference did not reach significance [85]. In a recent retrospective study Greenland and colleagues found a decrease in NK cell counts during acute AR and combined rejection and infection but an increase in NK cell counts in infection alone compared to healthy transplant recipients [25].

These findings are in line with newer research on NK cells: NK cells appear to be of pathogenic importance in kidney rejection by responding to missing host MHC ligands [87, 88] and in allograft tolerance by eliminating donor antigen-presenting cells [83, 89, 90]. Deficiency in NK cells has been shown to enhance AR, as detected in biopsies taken from mice [83]. According to a prospective study, NK cell numbers were raised in BAL fluid of stable lung transplant recipients and patients with BOS compared to non-transplanted individuals [91].

**Table:** 

Novel data identified NK cell count in BAL as a promising marker to assess lung transplant recipients. Patients with an episode of acute lung AR showed decreased numbers of NK cells. These data remain to be confirmed. Since NK cells are not measured in most lung transplant centres to date, clinical feasibility of such an assay has to be investigated.	

### B cells

B cells are lymphocytes expressing immunoglobulin on their surface, among other markers [92]. In their activated state as plasma cells B lymphocytes are the only producers of antibody molecules. Additionally, B cells activate T cells via antigen presentation [93] and organise the microarchitecture of lymphoid tissue [94, 95]. Recent data showed that B cells play an important role in the immunological response to an allograft, which goes beyond the production of antibodies [92, 96]. Indeed, studies on kidney rejection suggest an involvement of B cells in acute – T cell-mediated – AR [92, 97, 98]. Few data exist concerning the contribution of B cells to acute lung AR. In addition, diagnosis and definition of antibody-mediated rejection (AMR) is not well-defined in lung transplant recipients [99]. In the context of acute lung AR and BAL samples, increased numbers of B cells have been associated with acute AR [25, 85]. Most samples, however, contained less than 2 % of B cells, precluding further conclusions [25].

### Limitations

The studies included and discussed in our critical review have several limitations. Most studies have a retrospective design and report single centre experience [100]. Additionally, the methodological approaches and the patient cohorts are heterogeneous and vary substantially. Along this line, diagnostic criteria, immunosuppressive regimens, time points of sample collection and histological analyses and statistical evaluations differ between the studies.

More specifically, relying on one “unique” BAL cellular profile to diagnose acute AR is questionable since BAL cellular patterns differ according to the time-onset of acute AR episodes. As shown in the study by Tikkanen and colleagues, acute AR occurring early (1–180 days) after transplantation was associated with increased lymphocytes whereas elevated neutrophils were found in later-occurring rejection (>180 days after transplantation) [37].

In addition, many studies compared BAL and plasma samples to the histological pattern in TBB. However, whereas a direct relationship between the BAL profile and the severity of grade A (cellular) and B (lymphocytic bronchiolitis) rejection on concurrent TBB samples has been shown [41], some studies did not make a clear distinction between these grades. As illustrative example, Patil et al. merged grade A and B scores in their cumulative scoring system, while the semi-quantitative scoring system by Tikkanen and colleagues did not distinguish between inflammation of the alveolar and bronchiolar compartment [37, 44]. These approaches make it impossible to assess BAL cellularity according to separate grade A or B severity scores and to directly compare the results between both studies.

Furthermore, in most studies surrogate markers have been analysed separately, impeding further conclusions. Calculating a composite score, which takes into account several inflammatory cells, might provide further insights but is beyond the scope of this descriptive review. Taken together, these factors limit comparability, conclusions and direct translation of these findings in a clinical setting.

## Conclusions

Specific changes in differential cytology of BAL and plasma samples during acute lung AR have been identified. Data from blood analysis remain weak with only few studies assessing cytological changes in peripheral blood during acute AR.

The illustrative algorithm in Fig. 1 summarizes the most important findings of this review and assesses the likelihood of acute AR depending on blood and BAL analysis. A decline of FEV1 > 10 % should prompt imaging to exclude confounding factors such as pneumothorax or bronchial stenosis. Differential cellularity profile in BAL and, of lesser value, in blood samples might raise suspicion for the presence of acute AR. In detail, lymphocytosis > 20 %, neutrophilia ≥ 12 % without microbiological evidence for infection as well as the presence of eosinophils and basophils in BAL might provide hints for the presence of acute AR. The definite diagnosis of acute AR, however, still relies on lung biopsy.

BAL differential cytology might be more powerful in the context of CLAD than of AR since the presence of distinct cells (e.g. eosinophils) was found to be associated with worse outcome and shorter CLAD-free survival. Future research should, among others, focus on a composite score that includes several inflammatory cells in order to enhance the diagnostic value of BAL cell composition.

In conclusion, when used within the clinical context, BAL and serum samples might be useful to assist in decision-making and alter the likelihood for the presence or absence of acute lung AR. Of note, BAL and serum samples are no substitutes for transbronchial biopsies in the evaluation of lung function decline in lung transplant recipients.

## Abbreviations

AM, alveolar macrophages; AMR, antibody-mediated rejection; AR, allograft rejection; B cells, B lymphocytes; BAL, bronchoalveolar lavage; BOS, bronchiolitis obliterans syndrome; CLAD, chronic lung allograft dysfunction; IFN-γ, interferon gamma; IL, interleukin; IQR, interquartile range; MHC, major histocompatibility complex; NK cells, natural killer cells; NPV, negative predictive value; NRAD, neutrophilic reversible allograft dysfunction; PPV, positive predictive value; T cells, T lymphocytes; TBB, transbronchial lung biopsy; TNF-α, tumour necrosis factor alpha; PPV, positive predictive value; T cells, T lymphocytes; TBB, transbronchial lung biopsy; TNF-α, tumour necrosis factor alpha
